# The methylation of *SDC2* and *TFPI2* defined three methylator phenotypes of colorectal cancer

**DOI:** 10.1186/s12876-022-02175-3

**Published:** 2022-02-28

**Authors:** Ruixue Lei, Yanteng Zhao, Kai Huang, Qian Wang, Kangkang Wan, Tingting Li, Haijun Yang, Xianping Lv

**Affiliations:** 1 Department of Pathology, The Fourth Affiliated Hospital of Henan University of Science and Technology, Anyang Tumor Hospital, Anyang, Henan China; 2 Department of Transfusion, The First Affiliated Hospital of Zhengzhou University, Zhengzhou, 450052 China; 3 Department of Gastrointestinal Surgery, Department of General Surgery, The First Affiliated Hospital of Anhui Medical University, Hefei, China; 4 Department of Gastroenterology, Zhongnan Hospital of Wuhan University, Wuhan, Hubei China; 5 Wuhan Ammunition Life-Tech Company, Ltd., Wuhan, Hubei China

**Keywords:** Colorectal cancer, Methylation, Phenotypes, *SDC2*, *TFPI2*

## Abstract

**Background:**

Methylated *SDC2* and *TFPI2* are widely used for colorectal cancer (CRC) detection. However, they often miss some CRCs, which directly diminishes the sensitivity. Further investigations of the underlying mechanisms leading to the missed samples will facilitate developing more eligible methylation markers.

**Methods:**

CRC samples from TCGA and GEO datasets were divided into three groups, High-methylation/ High-methylation (HH), High-methylation/Low-methylation (HL), and Low-methylation/Low-methylation (LL) according to the methylation status of *SDC2* and *TFPI2* promoters. Variations in age, tumor location and microsatellite instable were then assessed between the three groups and verified in our custom cohort.

**Results:**

Samples of HL group preferred to derive from left-sided CRCs (*P* < 0.05). HH samples showed the highest microsatellite instability and mutation load (mean nonsynonymous mutations for HH/HL/LL: 10.55/3.91/7.02, *P* = 0.0055). Almost all mutations of *BRAF*, one of the five typical CpG island methylator phenotype (CIMP) related genes, were observed in HH group (HH/HL/LL: 51/0/1, *P* = 0.018). Besides, older patients were frequently found in HH group. Expression analysis identified 37, 84, and 22 group-specific differentially expressed genes (DEGs) for HH, HL, and LL, respectively. Functional enrichment analysis revealed that HH-specific DEGs were mainly related to transcription regulation, while LL-specific DEGs were enriched in the biological processes of extracellular matrix interaction and cell migration.

**Conclusions:**

The current study revealed that the performance of methylation-based markers might be affected by tumor location, patient age, mutation load and MSI, and these respective sides should be considered when developing new methylation markers for CRC detection.

**Supplementary Information:**

The online version contains supplementary material available at 10.1186/s12876-022-02175-3.

## Background

Colorectal cancer (CRC) is responsible for over 1 million new cases every year, and around 700,000 deaths occur worldwide, making it the third most frequently diagnosed cancer [1, 2]. In China, the incidence and mortality of CRC have been witnessed with an increasing trend of 12.8 in 2003 to 16.8 per 100,000 in 2011 and 5.9 in 2003 to 7.8 per 100,000 in 2011, respectively [3]. It is believed that CRC represents a heterogeneous group of tumors characterized by complex multifactorial phenotypes and multiple risk factors responses for the development of CRC [4, 5]. Many factors have been reported associated with CRC development, including unhealthy diets, smoking, overweight and obesity, inflammatory bowel disease and gut imbalanced microbiota [6–8]. Nearly half of CRCs were attributed to unhealthy diets such as low vegetable and fruit intake, high red and processed meat intake, and alcohol drinking in China in 2012 [7].

Syndecan-2 (*SDC2*), as one of the syndecan family of heparan sulfate proteoglycan, has been demonstrated to play an important role in cancer progression through regulation of cell adhesion, proliferation, and migration in many studies [9–12]. Tissue factor pathway inhibitor-2 (*TFPI2*) belongs to the Kunitz-type serine proteinase inhibitor family and is thought to be functional in regulating extracellular matrix digestion and re-modeling by inhibiting a broad spectrum of serine proteinases [13, 14]. Unlike the tumorigenic behaviors of *SDC2* in colon cancer cells, *TFPI2* has been shown as a tumor suppressor gene in several malignant tumors [15–18]. Interestingly, both promoters were frequently found hypermethylated in colon cancer cells compared to normal tissue cells in several epi-genomics studies [19, 20].

The frequently aberrant DNA methylation of *SDC2* and *TFPI2* makes them promise biomarkers for the early detection of CRC, and a few testing kits based on the two targets were also developed [19, 21–24]. However, the sensitivity of any single target (*SDC2* alone or *TFPI2* alone) did not exceed 90%. Several studies have suggested a better performance of combined multi-targets for CRC early detection than a single biomarker [25–27]. We observed an improved detection sensitivity when combining the two targets in our previous studies [28, 29], but not all CRC samples can be detected. During clinical practice, some CRC samples were detected only by a single or no target, reflecting the preference of different targets in distinguishing CRCs from normal samples, which directly affects the target utility.

Further investigations for the undetected CRC samples will help identify a small subset of tumors missed by the two targets and meanwhile benefit researchers to discover more eligible methylation markers. In this study, we first classified CRC samples into three methylator groups, *SDC2*/*TFPI2* double-positive group (HH, high-methylation/high-methylation), *SDC2*/*TFPI2* single positive group (HL, high-methylation/low-methylation), and *SDC2*/*TFPI2* double negative group (LL, low-methylation/low-methylation) according to the promoter methylation status of *SDC2* and *TFPI2*. The clinical-pathological parameters and molecular features were then evaluated by inner and outer cohorts, including TCGA, GEO, and our D311 CRC dataset.

## Methods

### Data preparation

The level 3 methylation data, raw read-count of RNA-seq, and clinical information of colon and rectum adenocarcinoma patients were retrieved from The Cancer Genome Atlas (TCGA) data portal (https://portal.gdc.cancer.gov/) by using the TCGAbiolinks R package [30]. The platform of methylation data from TCGA is Illumina Human Methylation 450 Beadchip (450 K array), and we also searched the Gene Expression Omnibus (GEO; http://www.ncbi.nlm.nih.gov/geo/) for eligible datasets that are generated by 450 K array. Two GEO datasets, GSE48684 [31] and GSE79740 [32] were then downloaded because of their available clinical information. Empirical Bayes (EB) batch adjustment along with a two-step quantile normalization method [33] was conducted for batch effect removal before GSE48684 and GSE79740 datasets were merged as one set. Missing values of the 450 k array were inferred and fulfilled by the Bayesian Network structure learning algorithm [34]. All samples without clinical information were removed. The preprocessed data used in this study is presented in Table 1.

**Table 1:** Clinical characteristics of subjects

Participant’s characteristics	TCGA CRC	GSE48684/GSE79740	D311 CRC
Normal	45	51	54
Tumor	394	108	257
Age, no. (%)			
≥ 60	254 (64.96%)	Not available	149 (58.75%)
< 60	137 (35.04%)	Not available	106 (41.25%)
Localization, no. (%)			
Left colon	148 (37.56%)	72 (66.67%)	82 (31.91%)
Right colon	147 (37.31%)	28 (25.93%)	49 (19.07%)
Rectum	46 (11.68%)	6 (5.56%)	90 (35.02%)
Others	53 (13.45%)	2 (1.85%)	36 (14.01%)
Gender, no. (%)			
Male	211 (53.55%)	Not available	152 (59.14%)
Female	183 (46.45%)	Not available	105 (40.86%)
MSI-H, no. (%)	65 (16.75%)	Not available	15 (8.06%)
MSS, no. (%)	323 (83.25%)	Not available	171 (91.94%)

### Patient samples collection

Fresh-frozen colorectal cancer tissues (n = 257) and colorectal mucosa (n = 54) tissues were collected at Zhongnan Hospital of Wuhan University at the time of surgery. All participants signed written informed consent, and their final diagnosis was determined based on colonoscopy or histological test. Participants who undertook any chemotherapy or radiotherapy or had incomplete information were excluded. The collected information includes age, gender, tumor size, tumor location, grade, and MSI status. Detailed demographic and clinical features of the participants are listed in Table 1. We classified rectosigmoid, descending colon, and splenic flexure tumors as left-sided cancer, whereas hepatic flexure and ascending colon tumors were grouped as right-sided cancer [35]. This study was approved by the medical ethics committee of Zhongnan Hospital of Wuhan University (No. 2019099).

### Identification of target regions

For *SDC2* and *TFPI2*, there are 32 and 23 probes located in their 5’UTRs and gene bodies in Illumina Human Methylation 450 k array, where 14 and 21 probes are within the two kilo-base upstream of transcript start site (TSS), respectively (Additional file 1: Table S1). We focused on the regions that showed the largest difference between normal and tumor samples to determine the appropriate target regions. First, the normal and tumor methylation values for each probe (β_Tumor_ and β_Normal_) were calculated using average β values of the normal and tumor samples in each dataset. Next, the probes with Δβ (β_Tumor_–β_Normal_) ≥ 0.3 were selected as the most varied probes. The threshold of 0.3 was chosen in this study as only about 5% of the top-ranked probes showed Δβ ≥ 0.3. Then, the most varied regions were identified according to the probe genomic coordinates. Three probes of *SDC2* that satisfied the criteria, cg16935295, cg04261408, and cg10292139, were found in a contiguous genomic position. Since adjacent CpG sites usually exhibited similar methylation status, also known as methylation block, the region consisting of four probes, cg16935295, cg04261408, cg14625631, and cg10292139, was finally selected as the target. The mean β values of these four probes were calculated as the methylation levels of *SDC2*. Similarly, we determined the region where seven probes, cg12973591, cg22799321, cg24531255, cg17338208, cg26739865, cg22441533, and cg14377593, located in as the target region of *TFPI2*, and their average β values were also calculated as the methylation levels of *TFPI2*.

### Identification of appropriate β threshold

In this study, the appropriate β threshold to define high-methylation or low-methylation was determined according to previous studies [36, 37]. Briefly, the distribution parameters of the methylation M-values were estimated by a Gaussian mixed model to determine the optimal M-value and the corresponding β-value. β-value and M-value are two metrics often used for the Infinium methylation array. β-value is more easily interpreted as an approximation of the overall methylation percentage at a given CpG site in a sample, while M-value is more statistically valid for the differential analysis of methylation levels. Although M-value is difficult to infer the methylation levels directly, it provides an insight into the distribution of methylation across the genome. Therefore, we investigated the distribution patterns of β-value and M-value of 450 K array on TCGA CRC dataset.

### Methylation-specific PCR

The genomic DNA was extracted by the UnigeneDx FFEE DNA extraction kit according to the manufacturer’s instructions. Tissue-derived genomic DNA was chemically modified by sodium bisulfite to convert unmethylated cytosine to uracil while leaving methylated cytosine unchanged. Methylation-specific PCR (MSP) was used to determine the methylation status of *SDC2* and *TFPI2* in normal and tumor tissue DNA, β-actin [38] was used as an internal control. Specific primers and probes for the target region of *SDC2* and *TFPI2* were designed as shown in Additional file 1: Table S2. We use the cycle threshold (Ct) value to determine the methylation status of these two genes, and the values for tissue samples were considered “invalid” if the ACTB Ct was greater than 36.00 and methylated *SDC2*/*TFPI2* were considered “detected” if the Ct values were less than 45.00. For samples with no amplification curve of the MSP occurred after 45 cycles, the Ct value was assigned 45.00. Three MSP replicates were conducted for each sample, and the average Ct value was used for further analysis.

### Identification of differentially expressed genes

Level-3 RNA-seq data of TCGA CRC cohort were preprocessed by removing zero expressed genes in more than 90% of all samples before performing differential expression analysis. DESeq2 [39] (V1.30.0) R package was used to perform a pairwise comparison between the three methylator groups to identify differentially expressed genes (DEGs). Genes with adjusted *P* values estimated by false discovery rate were recognized as candidate DEGs. Functional enrichment analysis of Gene Ontology and KEGG pathway was then conducted for the candidate DEGs using the web-based tool of GeneCodis [40].

### Statistical analysis

Comparisons for two paired or unpaired samples were performed for continuous variables using paired or unpaired student t-test. Kruskal–Wallis nonparametric analysis was used for multi-group comparisons of continuous variables. For categorical variables, fisher's exact test was applied to determine the non-random associations between SDC2/*TFPI2* methylator groups and clinical characteristics, such as age, sex, and tumor location. In this study, we chose 0.2 as the optimal β threshold. If the β values of *SDC2* and *TFPI2* promoters were > 0.2, they were defined as high-methylation, and vice versa as low-methylation. For Ct values, 38 was set as the threshold according to the previous study [41]. High-methylated *SDC2* or *TFPI2* were determined when their Ct values were < 38. Otherwise, they were determined low-methylated. All statistical analyses were performed using R software (version 3.6.0), and the source code was deposited to GitHub (https://github.com/amsinfor/methylator_group/blob/master/soure_code_v1.R).

## Results

### Methylation status of *SDC2* and *TFPI2* in CRC

**Fig. 1:**
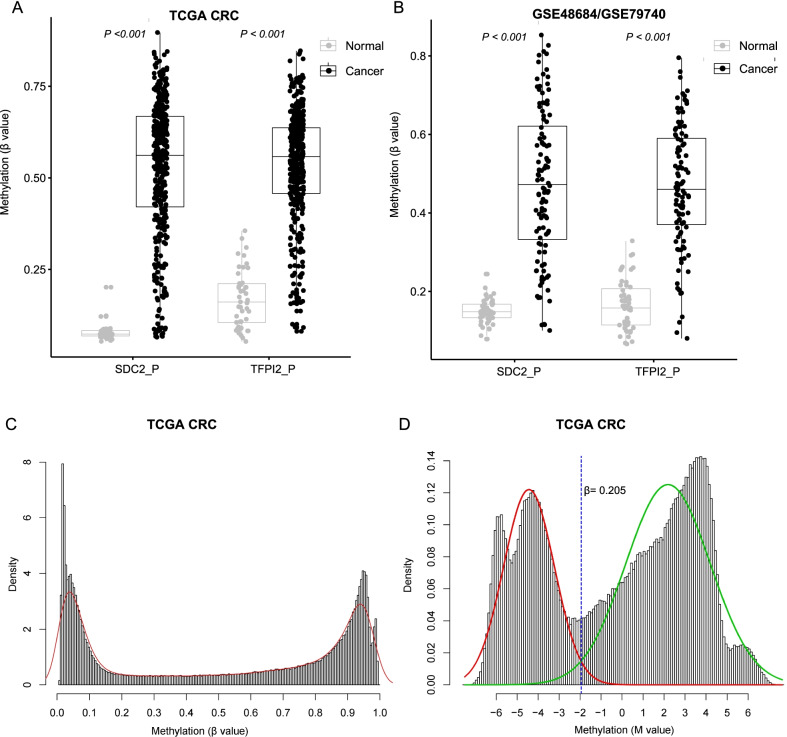
Methylation status of *SDC2* and *TFPI2* in TCGA cohort. **A**, **B** β values of *SDC2* and *TFPI2* in CRCs and normal controls in TCGA dataset (**A**) and GEO dataset (**B**). **C**, **D** The histograms and density distributions of methylation β-value (**C**) and M-value (**D**) on TCGA CRC. The red and green curves in panel D indicated the distribution of *M* values fitted by the Gaussian mixed model

We identified 4 and 7 probes in the promoters of *SDC2* and *TFPI2,* respectively (Additional file 1: Table S1). The average β values of these filtered probes were used as the methylation level of *SDC2* and *TFPI2* (herein termed as SDC2_P and TFPI2_P). Correlation analysis indicated that the methylation levels of both genes showed a weak negative correlation with the expression levels (Additional file 1: Figure S1). A lower Δβ value was found for *TFPI2* than *SDC2,* which might attribute to its higher background methylation level on normal controls (Fig. 1A, B). The β values were severely compressed around 0.05 and 0.95, and no obvious bimodal was observed (Fig. 1C). In contrast, the histogram of M values clearly showed a bimodal distribution (Fig. 1D). We attempted to estimate the distribution parameters of M values by fitting a Gaussian mixed linear model, and the results indicated that when M equaled to − 1.95, which corresponded to the β value of 0.205, the optimal boundary was determined between the two peaks (methylated peak and unmethylated peak). These findings implied that 0.2 could be an appropriate β threshold, which is consistent with the studies of Pan Du et al. and Sarah Dedeurwaerder et al. (see methods). We observed that high-methylated *SDC2* and *TFPI2* occurred in more than 85% of CRC samples in three datasets (average 88.21%). Approximately 10% of CRCs had single high-methylated gene (*SDC2* or *TFPI2*) (average 9.24%), and less than 3% of CRCs harbored low-methylated *SDC2* and *TFPI2* (2.56% on average) (Table 2).

**Table 2:** Methylation status of *SDC2* and *TFPI2* in three datasets

Methylation status	TCGA CRC	GSE48684/GSE79740	D311
High-M SDC2 and high-M TFPI2	353 (89.59%)	97 (89.81%)	219 (85.21%)
High-M SDC2 and low-M TFPI2	9 (2.28%)	3 (2.78%)	6 (2.33%)
Low-M SDC2 and high-M TFPI2	25 (6.35%)	5 (4.63%)	24 (9.34%)
Low-M SDC2 and low-M TFPI2	7 (1.78%)	3 (2.78%)	8 (3.11%)

### The association of methylator groups with tumor location

**Fig. 2:**
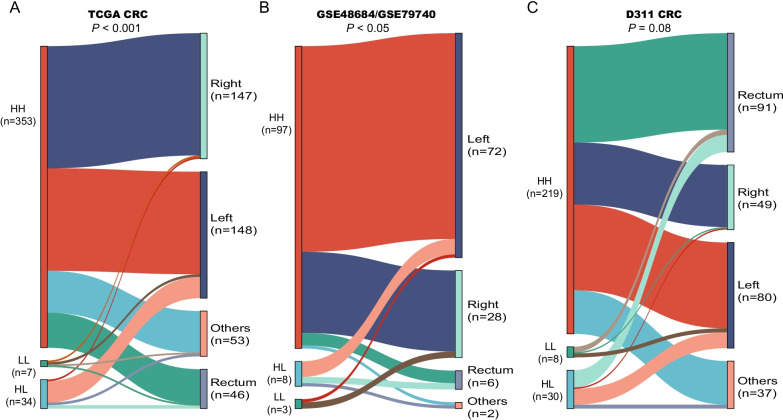
Sankey diagram showing the relationship between the 3 methylator groups and tumor locations in three datasets. **A**–**C** indicated the cohorts of TCGA CRC, GSE48684/GSE79740 and our custom cohort D311, respectively. Thickness of the connecting lines represented the number of samples on each group. *P* value was estimated by Fisher exact test

For early detection, HL and LL groups are very important because they can directly affect a given biomarker’s sensitivity. Therefore, we first compared the three methylator groups with tumor locations. Overall, no preference was observed for the distribution of HH group samples over different tumor locations in all three datasets, and this was similar for the LL group samples (Fig. 2A–C, Additional file 1: Table S3). Interestingly, the samples of HL group were more frequently originated from left-sided colon, while only a tiny fraction was from right-sided colon and rectum. These results indicated that the left-sided CRCs were more likely missed by the two targets, as the HL group samples preferred to locate in the left-sided colon.

### The association of methylator groups with genomic variations

**Fig. 3:**
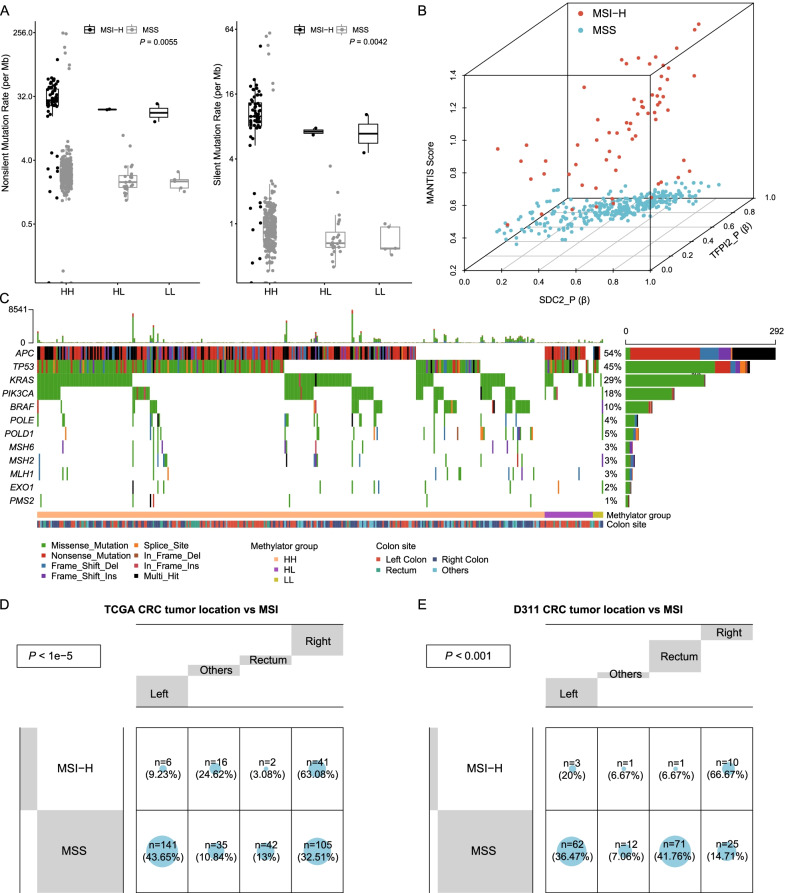
Genomic characteristics of three methylator groups in TCGA cohort. **A** Non-silent and silent mutation rates of HH, HL and LL groups. **B** The correlation of MANTIS score with β values of *SDC2* and *TFPI2* in TCGA CRCs. **C** Somatic mutation profile of five CIMP-related and MMR-related genes in HH, HL and LL groups. **D**, **E** Comparison of tumor location with MSI in TCGA CRCs (**D**) and D311 CRCs (**E**), significant p value is calculated using fisher exact test

**Fig. 4:**
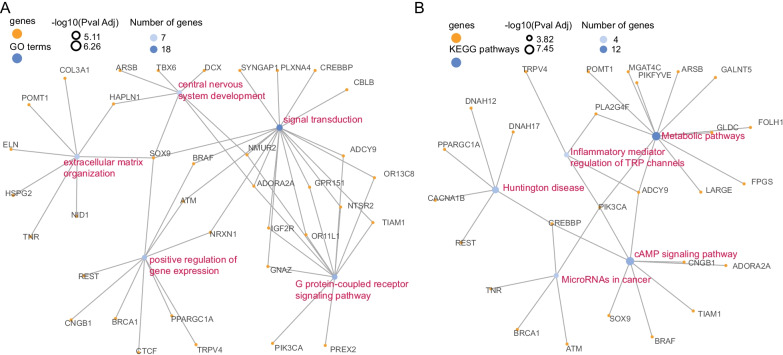
The top five enriched GO terms (**A**) and KEGG pathways (**B**). The yellow and blue points represented mutated genes and enriched terms, respectively. The point size indicated negative log10 of adjusted p values. The lables of GO terms and KEGG pathways were showing in red color

Microsatellite instability and hypermutation have been regarded as important molecular characteristics of CRCs. Compared to the other two groups, HH group CRCs presented the highest mutation load (Fig. 3A, *P* < 0.05) in the TCGA CRC dataset. According to the MANTIS scores [42], which is used to evaluate the MSI status, we grouped the TCGA CRCs into MSI-H if the score is > 0.4 and MSS if the score is ≤ 0.4. The β values of *SDC2* and *TFPI2* showed a high concordance with MANTIS scores in the MSI-H group (Fig. 3B, *P* < 0.001), which is consistent with the result that higher mutation load occurred in HH group. Meanwhile, the methylation levels of *MLH1,* which has been demonstrated its methylation associated with microsatellite instability [43], also showed significant variations between the three groups. Results indicated that HH group exhibited the highest methylation levels, followed by HL group and the lowest in LL group (Additional file 1: Figure S2). We studied the association of three methylator groups with the mutation of 5 typical CIMP-related genes, including *BRAF*, *PIK3CA*, *KRAS*, *TP53*, and *APC*. Almost all *BRAF*-mutated CRCs were in HH group (Fig. 3C, HH/HL/LL: 51/0/1, *P* = 0.018). We further compared the association between MSI status and tumor locations with TCGA CRCs and our D3111 CRCs. The MSI-H CRCs were preferred in the right-sided colon (Fig. 3D, E, *P* < 0.001), which possibly elucidated the potential mechanism that HL group CRCs were mainly in left-sided colon and MSI-H CRCs were less likely in HL group. Additionally, 111 mutated genes were significantly enriched between the three groups (Additional file 1: Table S4), involved in 53 biological processes and 31 KEGG pathways. The top 5 enriched terms were mainly related to signal transduction, expression regulation, and metabolic pathways (Fig. 4A, B).

### The association of methylator groups with patient age

**Fig. 5:**
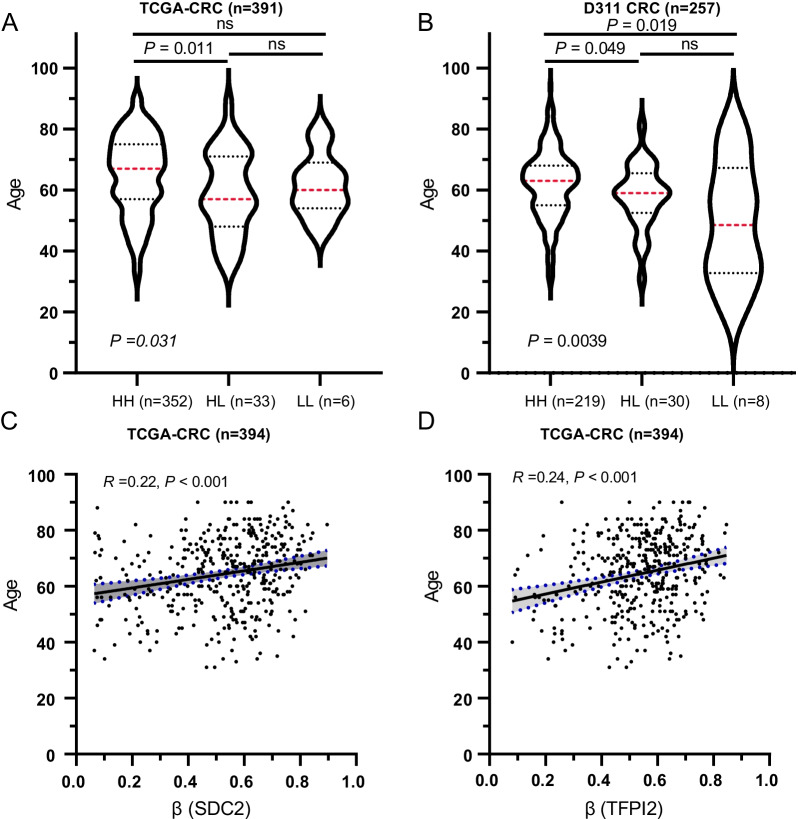
The association of patient age with methylation levels of *SDC2* and *TFPI2*. **A**, **B** Patient ages of HH, HL and LL groups in TCGA cohort (**A**) and D311 cohort (**B**). Patient age were not available for three samples in TCGA cohort. The dashed red line indicated median age. The upper and lower dashed lines represented 75% and 25% quantile. **C**, **D** The correlation of patient age with β values of *SDC2* and *TFPI2* in TCGA cohort. *P* value and correlation coefficient was calculated using *Pearsion*’ method

Patient age is one of the risk factors for colon cancer, and we found a significantly older age in HH group patients, while it was the youngest for LL group patients (Fig. 5A, B, *P* < 0.05). Since the genomic DNA methylation is associated with patient age, we observed a positive correlation of the methylation levels of *SDC2* and *TFPI2* with patient age (Fig. 5C, D). We then verified this result on the D311 dataset. Because of no direct correspondence between absolute Ct values and β values, we adopted a method similar to quantify gene expressions by quantitative PCR to determine the methylation levels of *SDC2* and *TFPI2*. The Ct values of internal reference gene, *ACTB*, were used as a control to quantify the relative methylation levels of these two genes, also called ΔCt. The 2^−ΔCt^ was then calculated for correlation analysis. Similar results were observed on the D311 dataset, though no strong correlation was presented (Additional file 1: Figure S3), indicating that young patients might be more likely to be miss-detected.

### Identification of DEGs among the three methylator groups

**Fig. 6:**
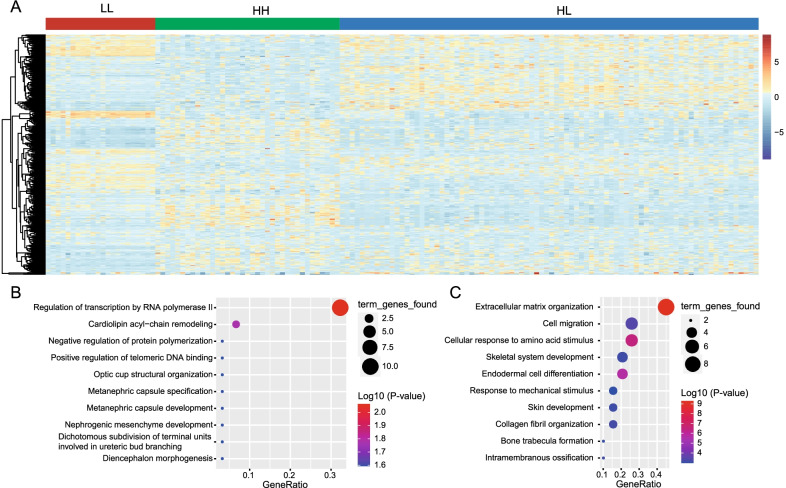
Identification of group-specific DEGs using the expression profiles of TCGA cohort. **A** The heatmap showing the expression profiles of HH-, HL- and LL-specific DEGs. The columns and rows represented group-specific DEGs and samples, respectively. **B** GO functional enrichment analyses for HH-specific DEGs. **C** GO functional enrichment analyses for LL-specific DEGs. The negative log10 (P value) of each term is colored according to the legend

We performed differential expression analysis using the gene expression profiles of TCGA CRC dataset to identify group-specific DEGs. DEGs among the three groups were first identified, and then their average expression levels were calculated separately for each group. The group-specific DEGs were defined as the genes in one group that presented the highest average expression values. A total of 37 HH-specific, 84 HL-specific, and 22 LL-specific DEGs were identified according to their average expression values on the three groups (Fig. 6A). Functional enrichment analysis indicated that HH-specific DEGs were mainly related to the regulation of transcription and other processes (Fig. 6B), while LL-specific DEGs are enriched in the biological processes of extracellular matrix interaction (ECM) and cell migration (Fig. 6C). No GO terms and KEGG pathways were significantly enriched for HL-specific genes. These results might elucidate potential alterations in the biological processes of ECM and cell migration that are related to the different characteristics of these three groups.

## Discussion

Quantifying aberrantly methylated genes was a feasible method for the early detection of CRCs. Several biomarkers have been demonstrated excellent performance in CRC early detection [24, 26, 27]. However, they often suffer the limitation with some positive samples miss detected, which directly affects their efficiency for CRC detection. In this study, we defined three CRC methylator groups, HH, HL, and LL, based on the methylation status of *SDC2* and *TFPI2* and then assessed their characteristics of genomic instability, mutation load, patient age, and biological processes. These findings suggested that it would be reasonable and essential to define three methylator groups according to the methylation status of these targets and will benefit guiding the development of more effective methylated biomarkers.

Our results revealed that high-methylated *SDC2* or *TFPI2* occurred in more than 95% of CRCs, suggesting that their methylation status can perform well to discriminate CRCs from normal controls. Previous studies have demonstrated the excellent performance of methylated *SDC2* and *TFPI2* alone or combined with other biomarkers for stool-based CRC detection [22, 44]. However, the combination of these two targets has not been reported. The present study revealed to some extent that the dual-target showed huge potential for CRC detection, which will be helpful for the development of stool-based noninvasive or blood-based minimally invasive detection techniques in the future.

However, in terms of a single target, nearly 10% of the samples had only one gene methylated, and here we defined them as HL group. Further analysis showed that more samples harboring high-methylated *TFPI2* and low-methylated *SDC2* were from HL group (about three times than the samples with high-methylated *SDC2* and low-methylated *TFPI2*). These results suggested that methylated *TFPI2* occurs more frequently on CRCs, implying that combining *TFPI2* with *SDC2* would help improve the sensitivity of CRC detection. Previous studies have demonstrated that multi-target outperformed single target [45, 46], evidenced by this study.

In clinical practice, the HL and LL group CRCs will cut down the sensitivity of these biomarkers. Our findings indicated that HL group CRCs more likely originate from the left-sided colon. Many differences were observed between the proximal (right-sided) and distal (left-sided) CRCs. For example, right-sided cancers were reported an increased incidence of proximal migration, while it was inversed for rectosigmoid tumors [47]. Moreover, the incidence between proximal and distal CRCs also differs in age and sex. Epidemiologic studies have revealed a higher incidence rate of distal CRC on males than females, and the proximal patients were older than distal patients, although increased ages were associated with the incidence rates of all subsites [48–50]. These data reflect extensive distinctions in molecular pathogenesis between the two anatomical locations, which might significantly impact tumorigenesis. Several studies have demonstrated that left-sided colon presents lower degrees of methylation than the right-sided colon, which was called the CpG island methylator phenotype, or CIMP, characterized by significant hyper-methylated CpG islands of tumor suppressors [51, 52]. Therefore, tumor location would be an important factor of biological heterogeneity. In this study, we observed lower methylation levels of *SDC2* in left-sided CRCs, and HL group CRCs were dominated by high-methylated *TFPI2* and low-methylated *SDC2*, which might explain why they appeared more frequently in the left-sided colon. These results suggested a potential impact of tumor locations on the early detection of CRCs when adopting abnormal methylated DNA as biomarkers.

Additionally, we found a positive correlation between the methylation levels of *SDC2* and *TFPI2* and MSI scores in MSI-H CRCs, as well as lower mutation load and rare *BRFA* mutations in HL group CRCs. Approximately 10% of CRCs harbor mutated *BRAF*, and its mutations are associated not only with poor prognosis but also with less benefit in metastatic CRCs when treated by antibodies [53]. Therefore, the methylator phenotypes may be a stratification factor in clinical therapies. It has been reported that CIMP tumors showed significant associations with *BRAF* mutations, MSI-H [52]. These results, on the other hand, confirmed that molecular events, such as epigenetic variations, instability, aberrant DNA mutations, and MSI, are coupled with each other.

Gene expression analysis identified methylator group-specific DEGs and functional annotation of LL-specific DEGs was suggested to focus on the biological process of ECM-receptor interaction, implying the potential alteration in molecular pathways in LL group CRCs. Interestingly, many studies have shown crucial roles of *SDC2* and *TFPI2* in the interaction of extracellular matrix with cell plasma [14, 54]. Besides, we also observed a significant enrichment of 111 mutant genes on GO term of extracellular matrix organization, which might, on the other hand, imply the tight association between ECM-receptor interaction and the three *methylator groups*. Overall, these findings revealed the possible impact of ECM process on the performance of methylated *SDC2* and *TFPI2* in detecting CRCs.

Colorectal cancer is a highly heterogeneous disease, often divided into proximal (right-sided) and distal (left-sided) cancer according to their anatomical locations. This classification is reasonable because of their distinctive embryonic derivation, the midgut and the hindgut for the proximal and distal colon, respectively [47, 55]. It might give rise to the altered methylations between the left- and right-sided colons, thus affecting the early detection of CRCs based on abnormally methylated DNA.

**Fig. 7:**
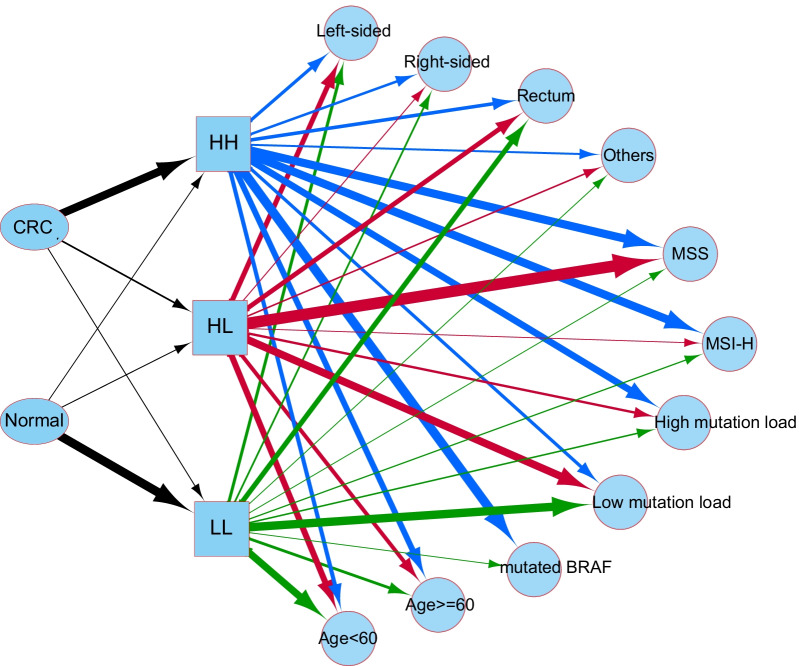
A diagram of possible associations of CIMP phenotype, tumor location, and MSI with the three methylator groups. The sample proportions of three methylator groups stratified by each clinical characteristic of CIMP phenotype, tumor location, and MSI were calculated separately according to the D311 cohort and used to draw this diagram. Ellipses and squares indicated the disease status and methylator groups. Circles represented the patient characteristics. The line width reflected the proportion of samples in a group that has a particular characteristic

In conclusion, the current study demonstrated the possible association of CIMP phenotype, tumor location, and MSI with the dual-target in CRC early diagnosis, making us propose a possible diagram of patient characteristics between the three groups (Fig. 7). In terms of genomic features, HH group CRCs are characterized by more often microsatellite instable (MSI), high mutation load, and frequent BRAF-mutated. HL group CRCs prefer to originate from the left-sided colon. Our observations suggested that the performance of methylation-based markers might be affected by tumor locations, CIMP phenotypes, patient age, mutation load, and MSI. This study also highlighted the different methylation status of *SDC2* and *TPFI2* between left and right-sided CRCs, which could lead to a detection bias in clinical practice as the right-sided tumors preferred to be detected by methylated *SDC2* and the left-sided by methylated *TFPI2*. Considering that left-sided CRCs are more prevalent in East Asian populations [56–58], the bias may enlarge the risk of CRC being missed when the two markers are applied for different ethnic groups. Therefore, examining physicians need to thoroughly evaluate the impact of multiple factors, including anatomical location, on a methylation-based test.

## Supplementary Information

**Additional file 1.** Supplementary Figures and Tables.

## Data Availability

The TCGA CRC 450k data and GEO datasets are publicly available online. The D311 data used and analyzed in study are available from the corresponding author on reasonable request.
