# Supplementary material for: The methylation of SDC2 and TFPI2 defined three methylator phenotypes of colorectal cancer
Source: BMC Gastroenterol. 2022 Feb 28;22:88. doi: 10.1186/s12876-022-02175-3 (PMC8886827; doi:10.1186/s12876-022-02175-3)
Supplement: Supplementary file 1 — Additional file 1. Supplementary Figures and Tables. [file 12876_2022_2175_MOESM1_ESM.docx]

The methylation of *SDC2* and *TFPI2* defined three methylator phenotypes of colorectal cancer

## Supplementary materials

## Supplementary figures

**Supplementary figure 1.** The correlation of methylation levels of *SDC2* and *TFPI2* with their expression levels. The *pearson’s* correlation coefficients and P-values were estimated.


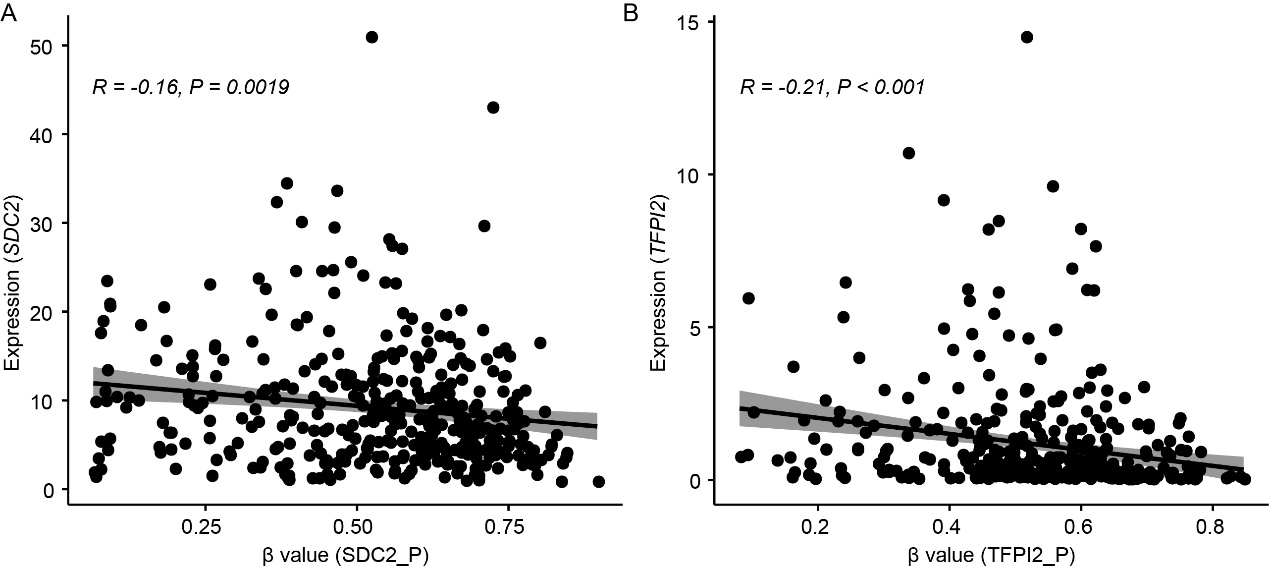


**Supplementary figure 2.** The methylation levels of *MLH1* promoter in three methylator groups. The error bars indicated mean β value±95% quantile.


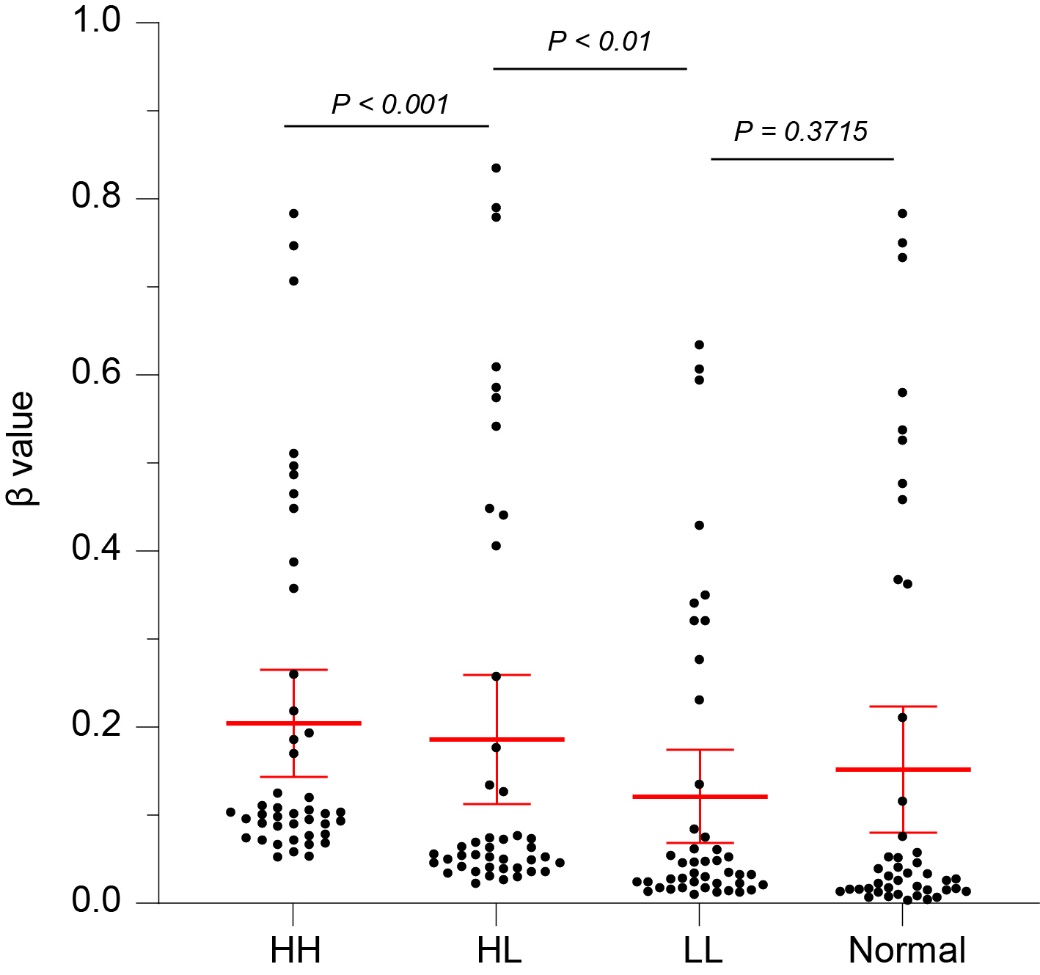


**Supplementary figure 3**. The correlation of patient age with the relative methylation levels of *SDC2* (A) and *TFPI2* (B) on D311 dataset. The *pearson* correlation coefficients and P-values were estimated.


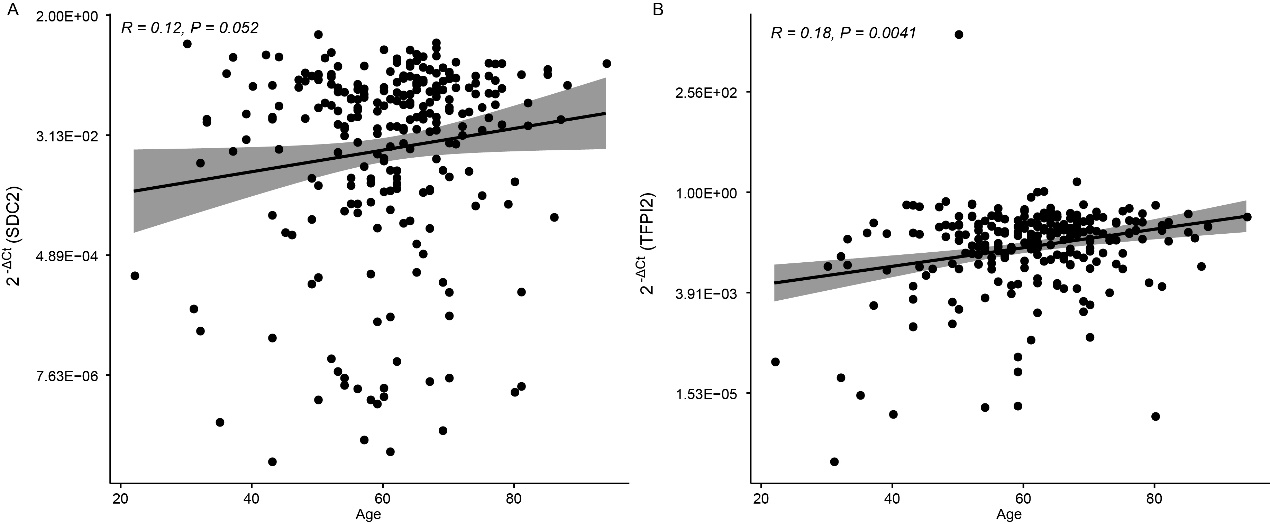


## Supplementary tables

**Supplementary table 1.** Probes identified in the promoter of *SDC2* and *TFPI2*.

| Symbol | Probes | Chr | Position | Located region | Mean β_N_ | Mean β_T_ | Δβ |
| --- | --- | --- | --- | --- | --- | --- | --- |
| *SDC2* | cg01329309 | 8 | 97574135 | TSS1500 | 0.769972 | 0.444852 | -0.32512 |
|  | cg26777303 | 8 | 97574567 | TSS1500 | 0.792445 | 0.839614 | 0.047169 |
|  | cg25664438 | 8 | 97574769 | TSS1500 | 0.091631 | 0.589168 | 0.497537 |
|  | cg23858558 | 8 | 97574776 | TSS1500 | 0.116948 | 0.342231 | 0.225283 |
|  | cg13096260 | 8 | 97574940 | TSS200 | 0.05634 | 0.52386 | 0.467521 |
|  | cg18719750 | 8 | 97574964 | TSS200 | 0.086139 | 0.37135 | 0.285211 |
|  | cg24732574 | 8 | 97574994 | TSS200 | 0.060108 | 0.348967 | 0.28886 |
|  | cg08979737 | 8 | 97575019 | TSS200 | 0.163213 | 0.422078 | 0.258865 |
|  | cg25070637 | 8 | 97575044 | TSS200 | 0.165667 | 0.383551 | 0.217884 |
|  | cg14538332 | 8 | 97575356 | 5'UTR;1stExon | 0.157773 | 0.437345 | 0.279571 |
|  | cg16935295 | 8 | 97575427 | 5'UTR;1stExon | 0.029983 | 0.512759 | 0.482776 |
|  | cg04261408 | 8 | 97575851 | 5'UTR;1stExon | 0.145016 | 0.720159 | 0.575143 |
|  | cg14625631 | 8 | 97576255 | 5'UTR;1stExon | 0.065955 | 0.326811 | 0.260856 |
|  | cg10292139 | 8 | 97576737 | 5'UTR;1stExon | 0.076813 | 0.547531 | 0.470718 |
|  | cg16673702 | 8 | 97576768 | Body | 0.076406 | 0.351991 | 0.275585 |
|  | cg07146119 | 8 | 97576771 | Body | 0.017442 | 0.304103 | 0.286662 |
|  | cg20594401 | 8 | 97576842 | Body | 0.032489 | 0.421707 | 0.389218 |
|  | cg03276479 | 8 | 97577134 | Body | 0.365371 | 0.529053 | 0.163683 |
|  | cg23356017 | 8 | 97579735 | Body | 0.871696 | 0.480532 | -0.39116 |
|  | cg27517823 | 8 | 97592266 | Body | 0.780612 | 0.586181 | -0.19443 |
|  | cg24862252 | 8 | 97605185 | Body | 0.889941 | 0.863061 | -0.02688 |
|  | cg10303967 | 8 | 97606894 | Body | 0.871102 | 0.500422 | -0.37068 |
|  | cg18030776 | 8 | 97613337 | Body | 0.918206 | 0.748643 | -0.16956 |
|  | cg14942501 | 8 | 97618935 | Body | 0.944924 | 0.920769 | -0.02416 |
|  | cg26842303 | 8 | 97633803 | Body | 0.87251 | 0.606306 | -0.2662 |
|  | cg15980656 | 8 | 97652412 | Body | 0.468614 | 0.463448 | -0.00517 |
|  | cg22137815 | 8 | 97654686 | Body | 0.876845 | 0.625905 | -0.25094 |
|  | cg14830748 | 8 | 97661375 | Body | 0.915412 | 0.868418 | -0.04699 |
|  | cg16962683 | 8 | 97671847 | Body | 0.924994 | 0.791127 | -0.13387 |
|  | cg14408978 | 8 | 97673223 | Body | 0.924742 | 0.915669 | -0.00907 |
|  | cg00446722 | 8 | 97689804 | Body | 0.909112 | 0.740917 | -0.16819 |
|  | cg12190613 | 8 | 97692186 | 3'UTR | 0.522147 | 0.358652 | -0.1635 |
| *TFPI2* | cg18024479 | 7 | 93357156 | Body | 0.41477 | 0.68104 | 0.26627 |
|  | cg19784477 | 7 | 93357337 | Body | 0.468614 | 0.463448 | -0.00517 |
|  | cg12973591 | 7 | 93357409 | 1stExon;5'UTR | 0.163575 | 0.611824 | 0.448249 |
|  | cg22799321 | 7 | 93357557 | 1stExon;5'UTR | 0.217003 | 0.618939 | 0.401935 |
|  | cg24531255 | 7 | 93357948 | 1stExon;5'UTR | 0.237853 | 0.561962 | 0.324109 |
|  | cg17338208 | 7 | 93357960 | 1stExon;5'UTR | 0.171085 | 0.607198 | 0.436113 |
|  | cg14775114 | 7 | 93357972 | 1stExon;5'UTR | 0.468614 | 0.463448 | -0.00517 |
|  | cg16934178 | 7 | 93358010 | TSS200 | 0.468614 | 0.463448 | -0.00517 |
|  | cg26739865 | 7 | 93358108 | TSS200 | 0.196212 | 0.589717 | 0.393505 |
|  | cg22441533 | 7 | 93358111 | TSS200 | 0.06139 | 0.267348 | 0.205959 |
|  | cg14377593 | 7 | 93358119 | TSS200 | 0.14252 | 0.487579 | 0.345058 |
|  | cg15649801 | 7 | 93358205 | TSS1500 | 0.278289 | 0.53481 | 0.256521 |
|  | cg10539069 | 7 | 93358211 | TSS1500 | 0.299769 | 0.545209 | 0.24544 |
|  | cg24084681 | 7 | 93358224 | TSS1500 | 0.273238 | 0.540124 | 0.266886 |
|  | cg07380959 | 7 | 93358259 | TSS1500 | 0.220239 | 0.556556 | 0.336317 |
|  | cg03333330 | 7 | 93358282 | TSS1500 | 0.301868 | 0.629139 | 0.327272 |
|  | cg09558850 | 7 | 93358381 | TSS1500 | 0.511149 | 0.736466 | 0.225317 |
|  | cg19854521 | 7 | 93358388 | TSS1500 | 0.543853 | 0.708855 | 0.165002 |
|  | cg13328713 | 7 | 93358463 | TSS1500 | 0.468614 | 0.463448 | -0.00517 |
|  | cg19103770 | 7 | 93358502 | TSS1500 | 0.468614 | 0.463448 | -0.00517 |
|  | cg18302726 | 7 | 93359029 | TSS1500 | 0.468614 | 0.463448 | -0.00517 |
|  | cg07603382 | 7 | 93359089 | TSS1500 | 0.468614 | 0.463448 | -0.00517 |
|  | cg27496965 | 7 | 93359132 | TSS1500 | 0.468614 | 0.463448 | -0.00517 |

Δβ=β_T_-β_N._ The 4 and 7 probes labeled in red bold were screened as the targets.

**Supplementary table 2.** Primers and probes used in this study

| Name | Primer/Probe sequences (5’- 3’) | Description | Length |
| --- | --- | --- | --- |
| SDC2_F | CGAGTTTGAGTCGTAATCGTTGC | MSP region 1 forward primer | 187 bp |
| SDC2_R | TCCGCCGACACGCAAACCACCAAACC | MSP region 1 reverse primer |  |
| SDC2_P | AACAAAACGAAACCTCCTACCCAAC | MSP region 1 probe |  |
| TFPI2_F | CGCGGAGATTTGTTTTTTGT | MSP region 2 forward primer | 163 bp |
| TFPI2_R | AACAAACATCGTCGCAAACCTC | MSP region 2 reverse primer |  |
| TFPI2_P | ATAAAACCCGACAAAATCCG | MSP region 2 probe |  |
| ACTB_F | CGCAATAAATCTAAACAAACTCC | ACTB forward primer | 89 bp |
| ACTB_R | AGGTTAGATGGGGGATATGT | ACTB reverse primer |  |
| ACTB_P | TCCCAAAACCCCAACACACT | ACTB probe |  |

**Supplementary table 3.** Samples in three methylator groups compared with tumor locations.

| Tumor location | TCGA CRC | | | GSE48684/GSE79740 | | | D311 | | |
| --- | --- | --- | --- | --- | --- | --- | --- | --- | --- |
|  | HH | HL | LL | HH | HL | LL | HH | HL | LL |
| Right-side | 143 | 2 | 2 | 26 | 0 | 2 | 47 | 1 | 1 |
| Left-side | 120 | 25 | 3 | 66 | 5 | 1 | 65 | 12 | 3 |
| Rectum | 41 | 4 | 1 | 4 | 2 | 0 | 73 | 14 | 4 |
| Other | 49 | 3 | 1 | 1 | 1 | 0 | 34 | 3 | 0 |
| Χ^2^ *P* | 0.00059 | | | 0.015 | | | 0.081 | | |

**Supplementary table 4.** Significantly enriched mutated genes between the three methylator groups.

| Symbol | Group1 | Group2 | n_mutated_group1 | n_mutated_group2 | p_value | fdr |
| --- | --- | --- | --- | --- | --- | --- |
| ATM | HH | HL+LL | 48 of 347 | 0 of 40 | 0.003679 | 0.04228 |
| DNAH17 | HH | HL+LL | 45 of 347 | 0 of 40 | 0.00535 | 0.04228 |
| DYNC2H1 | HH | HL+LL | 40 of 347 | 0 of 40 | 0.009907 | 0.042294 |
| MUC16 | HH | HL+LL | 105 of 347 | 5 of 40 | 0.011094 | 0.045163 |
| ATP10A | HH | HL+LL | 37 of 347 | 0 of 40 | 0.014274 | 0.046531 |
| SOX9 | HH | HL+LL | 53 of 347 | 1 of 40 | 0.014372 | 0.046531 |
| BRAF | HH | HL+LL | 51 of 347 | 1 of 40 | 0.017831 | 0.046531 |
| SCN9A | HH | HL+LL | 35 of 347 | 0 of 40 | 0.018175 | 0.046531 |
| MYO3A | HH | HL+LL | 35 of 347 | 0 of 40 | 0.018175 | 0.046531 |
| HERC2 | HH | HL+LL | 34 of 347 | 0 of 40 | 0.020497 | 0.046531 |
| DYNC1H1 | HH | HL+LL | 33 of 347 | 0 of 40 | 0.023109 | 0.046531 |
| MEGF8 | HH | HL+LL | 33 of 347 | 0 of 40 | 0.023109 | 0.046531 |
| IGF2R | HH | HL+LL | 32 of 347 | 0 of 40 | 0.026043 | 0.046531 |
| FMN2 | HH | HL+LL | 32 of 347 | 0 of 40 | 0.026043 | 0.046531 |
| USH2A | HH | HL+LL | 60 of 347 | 2 of 40 | 0.027886 | 0.046531 |
| TNR | HH | HL+LL | 31 of 347 | 0 of 40 | 0.02934 | 0.046531 |
| TIAM1 | HH | HL+LL | 31 of 347 | 0 of 40 | 0.02934 | 0.046531 |
| NIPBL | HH | HL+LL | 31 of 347 | 0 of 40 | 0.02934 | 0.046531 |
| CACNA1B | HH | HL+LL | 31 of 347 | 0 of 40 | 0.02934 | 0.046531 |
| DNAH12 | HH | HL+LL | 31 of 347 | 0 of 40 | 0.02934 | 0.046531 |
| SPTB | HH | HL+LL | 31 of 347 | 0 of 40 | 0.02934 | 0.046531 |
| CREBBP | HH | HL+LL | 30 of 347 | 0 of 40 | 0.033042 | 0.046531 |
| MAP2 | HH | HL+LL | 30 of 347 | 0 of 40 | 0.033042 | 0.046531 |
| PCDH10 | HH | HL+LL | 30 of 347 | 0 of 40 | 0.033042 | 0.046531 |
| A2ML1 | HH | HL+LL | 30 of 347 | 0 of 40 | 0.033042 | 0.046531 |
| AHNAK2 | HH | HL+LL | 44 of 347 | 1 of 40 | 0.03713 | 0.046936 |
| NRXN1 | HH | HL+LL | 29 of 347 | 0 of 40 | 0.037198 | 0.046936 |
| OTOGL | HH | HL+LL | 29 of 347 | 0 of 40 | 0.037198 | 0.046936 |
| HSPG2 | HH | HL+LL | 29 of 347 | 0 of 40 | 0.037198 | 0.046936 |
| PIK3CA | HH | HL+LL | 90 of 347 | 5 of 40 | 0.040969 | 0.046936 |
| FRYL | HH | HL+LL | 28 of 347 | 0 of 40 | 0.041862 | 0.046936 |
| LRRC7 | HH | HL+LL | 28 of 347 | 0 of 40 | 0.041862 | 0.046936 |
| PCDHA12 | HH | HL+LL | 28 of 347 | 0 of 40 | 0.041862 | 0.046936 |
| PRUNE2 | HH | HL+LL | 27 of 347 | 0 of 40 | 0.047095 | 0.047523 |
| MAP1B | HH | HL+LL | 27 of 347 | 0 of 40 | 0.047095 | 0.047523 |
| PLXNA4 | HH | HL+LL | 27 of 347 | 0 of 40 | 0.047095 | 0.047523 |
| PREX2 | HH | HL+LL | 27 of 347 | 0 of 40 | 0.047095 | 0.047523 |
| SCN2A | HH | HL+LL | 27 of 347 | 0 of 40 | 0.047095 | 0.047523 |
| ARID2 | HH | HL+LL | 27 of 347 | 0 of 40 | 0.047095 | 0.047523 |
| MTUS2 | HH | HL+LL | 27 of 347 | 0 of 40 | 0.047095 | 0.047523 |
| DIDO1 | HH | HL+LL | 27 of 347 | 0 of 40 | 0.047095 | 0.047523 |
| CDK5RAP1 | HL | HH+LL | 3 of 33 | 2 of 354 | 0.005045 | 0.04228 |
| NXF3 | HL | HH+LL | 3 of 33 | 4 of 354 | 0.01566 | 0.046531 |
| LAG3 | HL | HH+LL | 2 of 33 | 1 of 354 | 0.020069 | 0.046531 |
| OR11L1 | HL | HH+LL | 2 of 33 | 1 of 354 | 0.020069 | 0.046531 |
| ADORA2A | HL | HH+LL | 2 of 33 | 1 of 354 | 0.020069 | 0.046531 |
| KCNC1 | HL | HH+LL | 3 of 33 | 5 of 354 | 0.023601 | 0.046531 |
| GNAZ | HL | HH+LL | 3 of 33 | 5 of 354 | 0.023601 | 0.046531 |
| NID1 | HL | HH+LL | 4 of 33 | 11 of 354 | 0.030605 | 0.046531 |
| CYP4B1 | HL | HH+LL | 3 of 33 | 6 of 354 | 0.033351 | 0.046531 |
| HS3ST3B1 | HL | HH+LL | 3 of 33 | 6 of 354 | 0.033351 | 0.046531 |
| PLA2G4F | HL | HH+LL | 3 of 33 | 6 of 354 | 0.033351 | 0.046531 |
| ASTL | HL | HH+LL | 2 of 33 | 2 of 354 | 0.037995 | 0.046936 |
| NTSR2 | HL | HH+LL | 2 of 33 | 2 of 354 | 0.037995 | 0.046936 |
| SAP30BP | HL | HH+LL | 2 of 33 | 2 of 354 | 0.037995 | 0.046936 |
| AGBL1 | HL | HH+LL | 4 of 33 | 12 of 354 | 0.038357 | 0.046936 |
| DCX | HL | HH+LL | 3 of 33 | 7 of 354 | 0.044892 | 0.047523 |
| HAPLN1 | HL | HH+LL | 3 of 33 | 7 of 354 | 0.044892 | 0.047523 |
| GPR151 | LL | HH+HL | 2 of 7 | 2 of 380 | 0.001658 | 0.04228 |
| FPGS | LL | HH+HL | 2 of 7 | 3 of 380 | 0.002739 | 0.04228 |
| OR13C8 | LL | HH+HL | 2 of 7 | 4 of 380 | 0.004073 | 0.04228 |
| KIAA1958 | LL | HH+HL | 2 of 7 | 4 of 380 | 0.004073 | 0.04228 |
| EPS8 | LL | HH+HL | 2 of 7 | 4 of 380 | 0.004073 | 0.04228 |
| SMARCD3 | LL | HH+HL | 2 of 7 | 4 of 380 | 0.004073 | 0.04228 |
| TBX6 | LL | HH+HL | 2 of 7 | 4 of 380 | 0.004073 | 0.04228 |
| WDR33 | LL | HH+HL | 3 of 7 | 18 of 380 | 0.004209 | 0.04228 |
| UBE2K | LL | HH+HL | 2 of 7 | 5 of 380 | 0.005653 | 0.04228 |
| AP3B1 | LL | HH+HL | 2 of 7 | 6 of 380 | 0.007471 | 0.04228 |
| BICD1 | LL | HH+HL | 2 of 7 | 6 of 380 | 0.007471 | 0.04228 |
| ARSB | LL | HH+HL | 2 of 7 | 6 of 380 | 0.007471 | 0.04228 |
| UBQLN4 | LL | HH+HL | 2 of 7 | 6 of 380 | 0.007471 | 0.04228 |
| ELN | LL | HH+HL | 2 of 7 | 6 of 380 | 0.007471 | 0.04228 |
| LARGE | LL | HH+HL | 2 of 7 | 7 of 380 | 0.009522 | 0.04228 |
| KCNA10 | LL | HH+HL | 2 of 7 | 7 of 380 | 0.009522 | 0.04228 |
| INTS8 | LL | HH+HL | 2 of 7 | 7 of 380 | 0.009522 | 0.04228 |
| PRSS35 | LL | HH+HL | 2 of 7 | 7 of 380 | 0.009522 | 0.04228 |
| POMT1 | LL | HH+HL | 2 of 7 | 7 of 380 | 0.009522 | 0.04228 |
| GIF | LL | HH+HL | 2 of 7 | 7 of 380 | 0.009522 | 0.04228 |
| WFIKKN2 | LL | HH+HL | 2 of 7 | 7 of 380 | 0.009522 | 0.04228 |
| ABCG5 | LL | HH+HL | 2 of 7 | 7 of 380 | 0.009522 | 0.04228 |
| TUBGCP5 | LL | HH+HL | 2 of 7 | 8 of 380 | 0.011799 | 0.045163 |
| ZNF74 | LL | HH+HL | 2 of 7 | 8 of 380 | 0.011799 | 0.045163 |
| GALNT5 | LL | HH+HL | 2 of 7 | 9 of 380 | 0.014296 | 0.046531 |
| BRCA1 | LL | HH+HL | 2 of 7 | 9 of 380 | 0.014296 | 0.046531 |
| CHAF1A | LL | HH+HL | 2 of 7 | 9 of 380 | 0.014296 | 0.046531 |
| MGAT4C | LL | HH+HL | 2 of 7 | 10 of 380 | 0.017005 | 0.046531 |
| ATP9B | LL | HH+HL | 2 of 7 | 10 of 380 | 0.017005 | 0.046531 |
| WDR66 | LL | HH+HL | 2 of 7 | 11 of 380 | 0.019922 | 0.046531 |
| PCDHGB2 | LL | HH+HL | 2 of 7 | 11 of 380 | 0.019922 | 0.046531 |
| CBLB | LL | HH+HL | 2 of 7 | 11 of 380 | 0.019922 | 0.046531 |
| TRPV4 | LL | HH+HL | 2 of 7 | 11 of 380 | 0.019922 | 0.046531 |
| PPARGC1A | LL | HH+HL | 2 of 7 | 12 of 380 | 0.023039 | 0.046531 |
| TBC1D10B | LL | HH+HL | 2 of 7 | 12 of 380 | 0.023039 | 0.046531 |
| ASXL2 | LL | HH+HL | 2 of 7 | 13 of 380 | 0.026351 | 0.046531 |
| NMUR2 | LL | HH+HL | 2 of 7 | 13 of 380 | 0.026351 | 0.046531 |
| PIKFYVE | LL | HH+HL | 2 of 7 | 14 of 380 | 0.029852 | 0.046531 |
| KDM3B | LL | HH+HL | 2 of 7 | 14 of 380 | 0.029852 | 0.046531 |
| SYNGAP1 | LL | HH+HL | 2 of 7 | 14 of 380 | 0.029852 | 0.046531 |
| GLDC | LL | HH+HL | 2 of 7 | 14 of 380 | 0.029852 | 0.046531 |
| CNGB1 | LL | HH+HL | 2 of 7 | 15 of 380 | 0.033536 | 0.046531 |
| PCSK5 | LL | HH+HL | 2 of 7 | 15 of 380 | 0.033536 | 0.046531 |
| URGCP | LL | HH+HL | 2 of 7 | 15 of 380 | 0.033536 | 0.046531 |
| COL3A1 | LL | HH+HL | 2 of 7 | 16 of 380 | 0.037397 | 0.046936 |
| CTCF | LL | HH+HL | 2 of 7 | 16 of 380 | 0.037397 | 0.046936 |
| PHF3 | LL | HH+HL | 2 of 7 | 17 of 380 | 0.04143 | 0.046936 |
| REST | LL | HH+HL | 2 of 7 | 17 of 380 | 0.04143 | 0.046936 |
| ADCY9 | LL | HH+HL | 2 of 7 | 17 of 380 | 0.04143 | 0.046936 |
| FOLH1 | LL | HH+HL | 2 of 7 | 17 of 380 | 0.04143 | 0.046936 |
| KIAA1109 | LL | HH+HL | 3 of 7 | 44 of 380 | 0.041433 | 0.046936 |
| RAI1 | LL | HH+HL | 2 of 7 | 18 of 380 | 0.04563 | 0.047523 |
| N4BP2 | LL | HH+HL | 2 of 7 | 19 of 380 | 0.04999 | 0.04999 |
